# Radiometric Calibrations of Portable Sources in the Vacuum Ultraviolet

**DOI:** 10.6028/jres.093.004

**Published:** 1988-02-01

**Authors:** Jules Z. Klose, J. Mervin Bridges, William R. Ott

**Affiliations:** National Bureau of Standards Gaithersburg, MD 20899

**Keywords:** arc (argon), arc (blackbody), arc (hydrogen), irradiance, lamp (deuterium), radiance, radiometry, Standards (radiometric), ultraviolet, vacuum ultraviolet

## Abstract

The radiometric calibration program carried out by the vacuum ultraviolet radiometry group in the Atomic and Plasma Radiation Division of the National Bureau of Standards is presented in brief. Descriptions are given of the primary standards, which are the hydrogen arc and the blackbody line arc, and the secondary standards, which are the argon mini- and maxi-arcs and the deuterium arc lamp. The calibration methods involving both spectral radiance and irradiance are then discussed along with their uncertainties. Finally, the calibration services are delineated in an appendix.

## 1. Introduction

The vacuum ultraviolet (VUV) region of the spectrum has become important in several areas of research and development. These include space-based astronomy and astrophysics, thermonuclear fusion research, ultraviolet laser development, and general atomic physics research. Applications of VUV radiation in chemical, biophysical, and medical fields are widespread. Many applications require knowledge of not only the wavelength of the radiation involved but also the intensity or flux of radiation. This implies a calibration of some type. The calibration may be based upon a standard source, i.e., one whose output is known, or a standard detector, i.e., one whose response to a given radiation level is known. Two general cases can be distinguished. In the first case one wishes to determine how much radiation a source such as the sun or a plasma device is emitting at a given wavelength. Usually, the source is not monochromatic, so a monochromator must be used to select the desired wavelength. In this case the most direct procedure is to employ a standard source. The source to be investigated as well as the standard source are set up in turn so that radiation from each source passes through the same monochromator and optical elements. The calibration is performed essentially by a direct substitution of the standard source for the one to be calibrated.

The second case occurs when one wishes to know the flux in a monochromatic beam of radiation, such as the flux emerging from the exit slit of a monochromator. For this determination a standard detector is more appropriate; the flux is determined by simply measuring the signal when the detector is irradiated with the beam to be calibrated. If one were to attempt to perform the calibration in the first case above using a standard detector or in the second case using a standard source, one would in both cases need to know the spectral efficiency of the monochromator used in the measurement. This would require an additional measurement using a second monochromator and would introduce additional uncertainties and complexities. Therefore, a need exists for both standard sources and standard detectors.

Standard sources may be divided into primary standards and secondary or transfer standards. Primary standards are ones whose output is known from basic principles. The primary standards of VUV radiation include plasma sources, especially the wall-stabilized hydrogen and blackbody line arcs, and electron storage rings emitting synchrotron radiation.

There are storage rings at several laboratories, including NBS, which are used as primary VUV radiation standard sources. They produce highly-polarized continuum radiation for wavelengths ⪆0.03 nm. Limited access to storage rings and problems with the emitted polarized light, however, make it desirable to have available other standard VUV sources. The wall-stabilized hydrogen and blackbody line arcs were developed to provide alternative primary standard sources. These sources, however, are also not able to be easily used for calibrations. Hence, secondary or transfer standards which are relatively easy to apply have been developed. These include the deuterium lamp and the argon mini-arc. These sources are more readily available and make possible relatively inexpensive and convenient calibrations. Also for researchers having access to a storage ring, the secondary standards are useful in making possible more frequent calibrations. Finally, the secondary standards possess some useful properties not characteristic of the available primary standards such as, for example, emission over a relatively large solid angle.

The two principal radiometric quantities which are measured and calibrated are *radiance* and *irradiance.* For an object or source which emits radiation, the *radiance* is the radiant power emitted per area per solid angle, *L* = (W cm^−2^ sr^−1^). If the source emits a continuum, i.e., emits radiation at all wavelengths near a particular wavelength, the *spectral radiance* is the radiance per wavelength interval or bandpass, L*_λ_*=(W cm^−2^ sr^−1^ nm^−1^). The radiance will in general vary over the source area, the direction, and the wavelength. The definition assumes that the area, solid angle, and wavelength band are small enough that the radiance does not vary greatly within these quantities. Irradiance is the radiant power incident upon a target per area, *E* = (W cm^−2^), and *spectral irradiance* is the radiant power incident upon a target per area per wavelength band, *E_λ_*=(W cm^−2^ nm^−1^). A source of radiation may serve as a standard source of irradiance by operating it at a given distance from the target area. Some sources may be used as either standard radiance or standard irradiance sources. A separate calibration must be performed, however, for each quantity.

The services performed by the Atomic and Plasma Radiation Division of the National Bureau of Standards include tests and calibrations of portable secondary VUV standard radiance and irradiance sources. These are usually rare-gas dimer lamps, which emit continuum radiation over limited wavelength ranges, and hollow cathode lamps, which emit spectral lines in the wavelength range from the VUV through the visible. All sources are generally supplied by customers. The main groups of customers have included those in the fields of space-based astronomy and solar physics who have used standard sources to calibrate satellite, rocket, or balloon-borne spectrometers. Other customers have needed calibrations in the 100–300 nm range for plasma radiation studies.

## 2. Apparatus

### 2.1 Primary Standards

#### 2.1.1 The Hydrogen Arc

**Figure 1:**
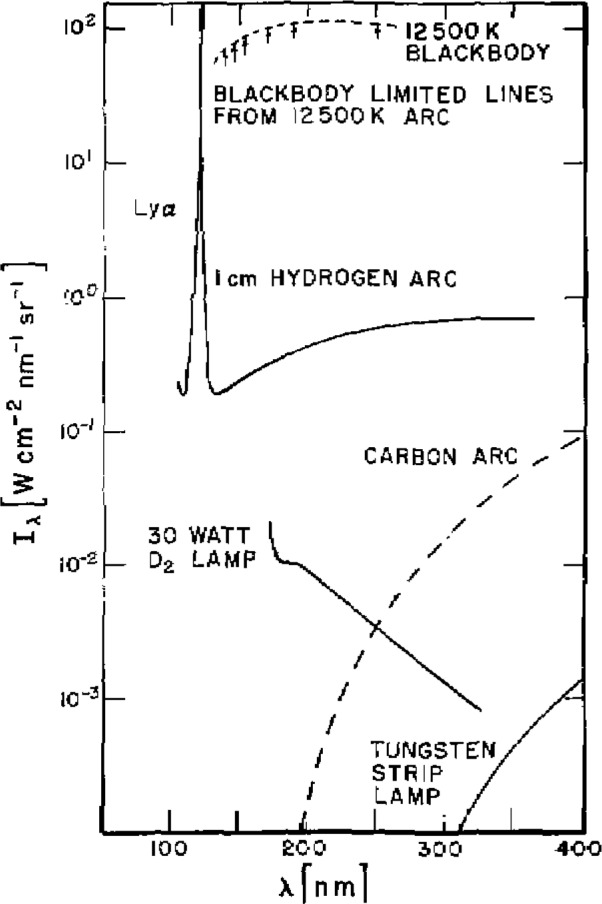
Comparisons of spectral radiances for far UV sources. The output of the hydrogen arc is given for the temperature of maximum continuum emission; the outputs of the other sources are at typical operating conditions.

A high temperature wall-stabilized steady-state hydrogen arc has been developed as our primary standard of spectral radiance [1]. This type of arc lends itself to such a use because at sufficiently high temperatures it yields absolute intensities independent of other radiometric standards or of the accuracy of any plasma diagnostics. Previous efforts at lower powers were hindered by large uncertainties in plasma diagnostics, a difficulty that has been overcome in the high temperature arc. Figure 1 illustrates the UV spectrum of several of the more common standard sources, including that part of the hydrogen arc spectrum that is the subject of this paper.

**Figure 2:**
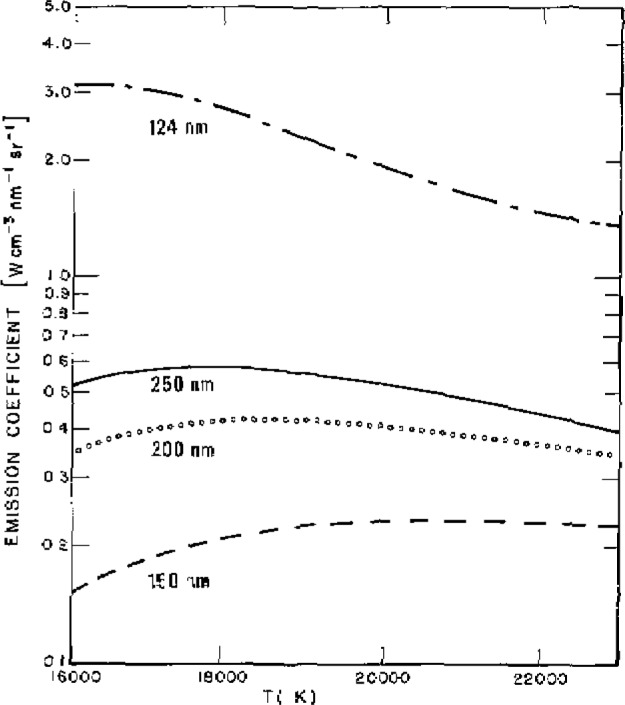
The emission coefficient for a 1-atm hydrogen plasma in LTE as a function of temperature for several wavelengths; 250 nm (—); 200 nm, (○○○○); 150 nm (----); and 124 nm (— - — -).

The method depends upon the phenomenon that the continuum emission coefficient for a strongly ionized hydrogen plasma which is in or close to the condition of local thermodynamic equilibrium (LTE) is calculable to within one percent [2]. This follows from the fact that the essential spectroscopic constants, i.e., the continuum absorption coefficients and transition probabilities, are exactly known for atomic hydrogen. The continuum intensities emitted from a typical pure hydrogen wall-stabilized arc discharge in the spectral region above 91.5 nm are optically thin and a function of the electron density and temperature [3,4]. In the low power hydrogen arc the electron density and temperature are determined from plasma diagnostics in the visible spectral region using available radiometric standards [3]. These quantities are then used to calculate the continuum intensities in the VUV. There are known to be significant uncertainties in the plasma diagnostics, and as a result we have adopted as our primary standard a higher power hydrogen arc which operates such that a 2-mm diameter wall-stabilized discharge reaches a temperature of about 20,000 K. For these conaditions the continuum emission coefficient as a functicn of temperature shows a broad maximum which shifts with wavelength as is shown in figure 2. This optimum condition is brought about essentially by the compensating effects of an increase in the ionization fraction and a decrease in the total number density as the temperature is increased in a constant 1-atm pressure operating environment. Beyond this maximum [5,6] the ionization is practically complete, and any further increase in arc temperature results only in a gradual decrease in intensity because of the decrease in the number density. The weak wavelength dependence of this maximum seen in figure 2 occurs because of the change in the energy distribution of the equilibrated electrons as the electron temperature is varied.

**Figure 3:**
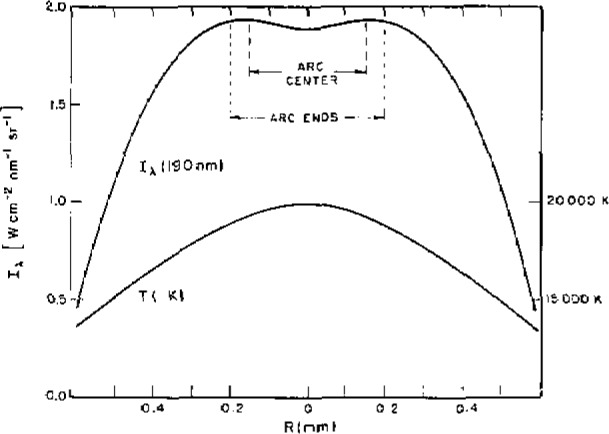
Radial dependence of the continuum intensity and arc temperature for a 2-mm wall-stabilized hydrogen arc operating at 80 A. The dashed lines define the dimensions of the plasma that is observed by spectrometers.

Figure 3 shows the measured radial dependence of the hydrogen continuum emission coefficient at 190 nm and the corresponding calculated LTE temperature [2] for an arc current of 80A. Because the maximum in the coefficient is broad and the absolute magnitude of the peak intensity is not very sensitive to the electron temperature, the emission characteristics of the plasma are nearly homogeneous over its central region which extends to about 0.6 mm from the arc axis. This is especially significant if only a small sample of the region is observed as indicated in the figure. First, it means that the alignment precision, which was responsible for much of the uncertainty in the low power arc method, is not critical here. Second, it means that the arc current necessary to obtain the maximum emission coefficient is also not critical.

Additional advantages of operating the hydrogen arc under such conditions are (1) the H_2_ Lyman band molecular emission, which limited the low power hydrogen arc to wavelengths longer than 165 nm, is negligible; (2) the assumption of LTE appears to be very closely fulfilled as shown by experimental consistency checks and theoretical validity criteria [7]; and (3) the hydrogen arc as it is described here constitutes a true primary standard of spectral radiance in that except for the minor uncertainties associated with the theoretical model for the plasma, knowledge of the absolute continuum intensities does not depend on any other standards of calibration or sophisticated plasma diagnostics and requires only a measurement of the ambient pressure (≈ 1 atm) and the arc length. The procedure for applying the arc as a radiometric standard involves only adjusting the arc current for maximum signal at the wavelength of interest. The overall system efficiency is given then by the ratio of the detector response to the hydrogen arc spectral radiance calculated using the maximum LTE emission coefficient at 1-atm pressure and the actual physical length of the discharge.

Figure 1 shows the wavelength dependence of the maximum emission coefficient of the hydrogen arc plasma, and table 1 lists the values of the same quantity obtained through a calculation of the hydrogen continuum [2]. The Stark broadened wing of the Lyman α line at 122 nm has been included in these calculations and, as can be seen from the table, becomes significant at wavelengths below 140 nm [8]. The uncertainty in the calculated continuum emission coefficient is estimated to be about 2%, due mostly to uncertainties in the high density plasma corrections [2]. Combining this uncertainty in quadrature with the uncertainties in the Stark broadening calculations, the uncertainty in the total emission coefficient comes out to be 2% above 140 nm, 8% at 130 nm, and 13% at 124 nm. These uncertainties and all those succeeding unless stated otherwise are taken to be 2 standard deviations (2*σ*), i.e., as having a confidence limit of about 95%.

**Figure 4:**
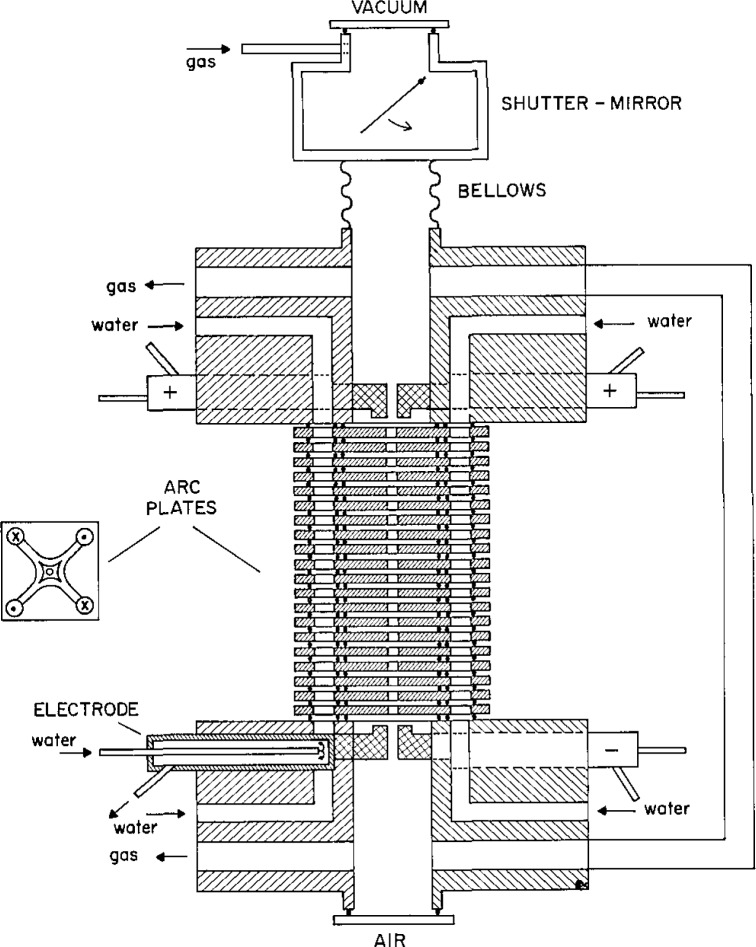
Schematic of the hydrogen wall-stabilized arc. A cutaway top view drawing of one of the arc plates illustrates the flow of cooling water through specially machined channels within each plate. The symbols in the four corners show the direction of water flow.

Three factors are of special concern in applying the high power hydrogen arc standard: the arc length, spatial resolution, and off-axis H_2_ emission. Figure 4 is a schematic of the hydrogen arc (1,4,9]. The arc is struck between a set of four tungsten anodes and four tungsten cathodes that are symmetrically located and share the current load equally. At each end of the 2-mm diameter channel formed by the 20 water-cooled stacked copper plates, the discharge expands and separates into four smaller arcs terminating at each of the electrode tips. The length of this inhomogeneous region is made small compared to the total length of the arc, and the emission coefficient and temperatures are lower than in the channel. Therefore, its contribution to the total arc signal obtained in an end-on measurement along the axis of the discharge is small but not precisely known. The minimum possible length of the arc is 5.05 cm, the total length of the channel formed by the 20 arc plates, and the maximum length is 5.5 cm, the total distance between the electrodes. The estimated length of the homogeneous hydrogen arc plasma was thus taken to be 5.3 cm with an uncertainty of 5% (2*σ*).

Spatial resolution is not much of a problem for the high power arc since the emission characteristics are nearly uniform in the vicinity of the arc axis. The regions of the arc that fall into the cone of observation determined by the f/200 aperture and the 0.30 mm field stop of the observing optics are illustrated in figure 3. Thus, knowledge of the emission coefficient as a function of radial position allows the calculation of the effect of spatial averaging. The main result is that the integrated emission coefficient $\bar{\epsilon }$ has a maximum about 1% lower than ϵ_max_ from a truly homogeneous plasma with an axis temperature $T(\bar{\epsilon })$ slightly higher than T(ϵ_max_). The arc plasma is cylindrically symmetric, and because the area of the differential shells increases as 2*π*rΔr, the off axis regions are weighted more than the axis region. Therefore, when one obtains maximum signal, the current has been adjusted so that the temperature maximum occurs slightly off-axis as seen in figure 3.

**Figure 5:**
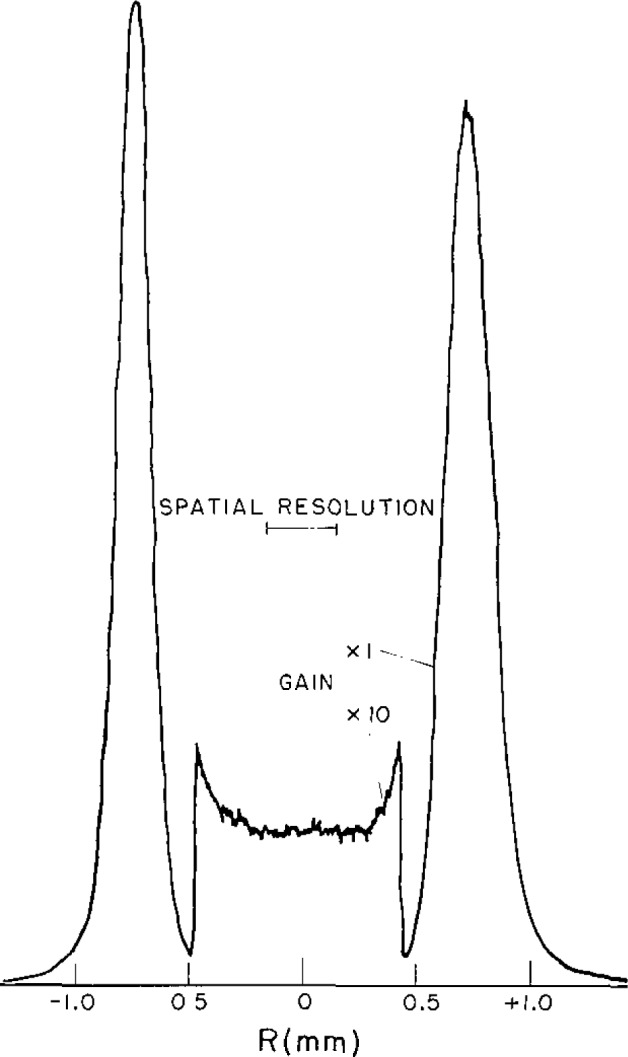
The radial dependence of the hydrogen arc spectral radiance at 160.6 nm for an arc axis temperature of about 19,500 K. The intense off-axis radiation is due to Lyman band molecular emission. The nearly uniform radiation in the vicinity of the axis contains no molecular contributions, but is due to the hydrogen continuum that is being used as a spectral radiance standard.

Below 165 nm the Lyman bands of H_2_ are emitted from low temperature off-axis regions. Figure 5 shows a radial scan of the arc at a wavelength where one of the stronger H_2_ features can be observed. The off-axis line peak is about 100 times larger than the on-axis hydrogen continuum, but clearly if the observation column is restricted to no more than the central 0.5 mm diameter plasma, H_2_ lines should not be observed. Molecular emission from the inhomogeneous end regions also is a possibility. However, none is observed since the arc terminates at points that are out of the line of observation (the cooler plasma regions are off-axis) and the end-layer on-axis temperature gradient is quite sharp (the length of the cool plasma is therefore very short).

**Figure 6:**
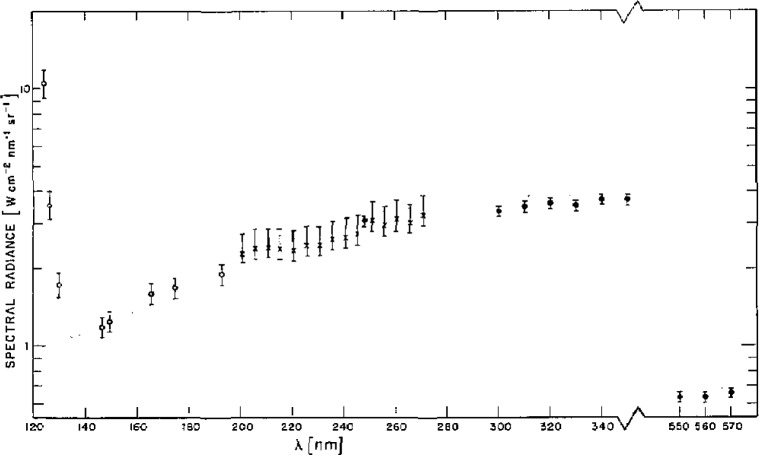
Maximum spectral radiance vs wavelength calculated for a 5.3-cm length hydrogen arc plasma in LTE at 1 atm pressure. The calculation is shown as a grid and represents the rms uncertainty due to uncertainties in the arc length (±5%) and the calculated emission coefficient (between 2% and 13%). The points are actual measurements of the spectral radiance calibrated either with a tungsten strip lamp (·), blackbody limited lines (○), or a low power hydrogen arc (×).

In figure 6 the calculated spectral radiance of the high power hydrogen arc is compared with calibrations of that arc made with several other standard sources [1]. The theoretical spectral radiance is the product of the theoretical maximum hydrogen plasma emission coefficient and the arc length. This quantity is graphed as a shaded area which represents the rms uncertainty due to the previously described uncertainties in both the calculated emission coefficient and the arc length. The solid and open circles represent the measured continuum intensities using, respectively, a calibrated tungsten strip lamp [10] and blackbody limited lines from another arc for spectral radiance normalization. The crosses represent the measured intensities using the low power hydrogen-arc standard. The error bars attached to the data points represent uncertainties associated not with the hydrogen arc but with the various standards used to calibrate the spectral radiance. The discontinuity in the wavelength scale is due to the interruption of the hydrogen spectrum by the Balmer line series of atomic hydrogen, which dominates the high temperature arc spectrum above 360 nm except for a small region around 560 nm between H*_α_* and H*_β_*. Inspection of figure 6 leads us to conclude that the various standards are consistent with one another over the entire range of comparison.

In summary, a high power atmospheric pressure hydrogen arc capable of operating at temperatures on the order of 20,000 K has been examined theoretically and experimentally and found to be suitable as a primary radiometric standard in the VUV. The experimental investigations have shown that at such temperatures molecular hydrogen emission at wavelengths shorter than 165 nm is negligible, and the hydrogen plasma continuum emission coefficient can be measured throughout the 124–360 nm spectral range. By operating the arc at currents such that the maximum emission coefficient is reached, the uncertainties associated with various plasma diagnostics and alignment imprecisions are minimized to the one-percent range. The results of a comparison with other available standard sources are consistent with the estimated 2*σ* uncertainty of ±5% in the hydrogen arc spectral radiance between 140 and 360 nm. This uncertainty is due mainly to the uncertainty in the measurement of the arc length. Below 140 nm the Lyman *α* Stark broadened wing becomes significant, and the estimated uncertainty in the hydrogen arc intensities increases to about ±9% at 130nm and ±14% at 124 nm due mainly to uncertainties in the plasma line broadening theory. These results are also consistent with a comparison standard. Finally, all experimental comparison data confirm that the hydrogen arc spectral radiance is calculable throughout the 124–360 nm range without dependence on any other radiometric standard or plasma diagnostic technique. Therefore, the high power hydrogen arc is a true primary standard. A detailed description of the hydrogen arc is given elsewhere [4].

#### 2.1.2 Blackbody Line Arc

The short wavelength limit of the hydrogen arc at about 124 nm is determined by the onset of the Stark-broadened, optically thick Lyman line series of atomic hydrogen that dominates the spectrum between 94 and 124 mm. Since the short wavelength cutoff of magnesium fluoride windows which are used in many VUV instruments is at about 115 nm, it would be convenient to have a primary standard which covers the range 115 to 124 nm. This need has been met with the development of the blackbody line arc [11–13].

Blackbody radiances for a number of prominent ultraviolet spectral lines are determined in the following manner. A wall-stabilized steady-state arc source is operated at about 12,000 K in argon with small admixtures of oxygen, nitrogen, and carbon dioxide. The admixtures produce very strong emission for their principal resonance lines, which all happen to lie conveniently in the wavelength range from 115 to 250 nm. These extremely strong resonance lines reach the blackbody intensity limits even at very small concentrations of the elements in the plasma, i.e., they become “optically thick.” More precisely, the central regions of these line profiles reach, for a small wavelength range of a few hundreths of a nanometer, the intensity a blackbody would have at the arc temperature. Thus, the arc represents, for a few narrow wavelength bands in the UV spectrum, a very high temperature blackbody. To utilize this arc as a standard source, the temperature must be accurately determined using standard plasma spectroscopic techniques including absolute radiometry in the visible region of the spectrum. Subsequently, using Planck’s radiation law, absolute intensities are established for these narrow spectral bands and are utilized as calibration points. Calibrations at other wavelengths are usually found by interpolation.

This technique has been used in a number of experiments and has been found to be reliable, with uncertainties ranging from about 10% at 250 nm to about 25% at 115 nm. For general use, however, it has the significant deficiency that large areas of the far UV have to be covered by interpolation since very few blackbody limited lines are found outside the ranges 115 to 126 nm and 146 to 175 nm. Since we are primarily interested in the range 115 to 124 nm, this is no great handicap here.

The accuracy of the blackbody line method depends critically on the measurement of the arc temperature, which is applied in the Planck function to determine the intensity of the blackbody ceilings of the optically thick resonance lines. It has been estimated that no single spectroscopic method is sufficient to determine the temperature of such a plasma with small O_2_, N_2_, and CO_2_ additions to within an uncertainty of ±2% [11]. This represents an uncertainty in the blackbody ceiling of about 10% at 250 nm and about 25% at 115 nm as was mentioned above. To reduce this uncertainty, it was recommended that several methods be applied, and the results averaged. However, a single method, which is thought to be superior, was adopted here to determine the blackbody temperature.

The blackbody ceiling of the CI emission line at 247.9 nm is measured with a UV-calibrated tungsten strip lamp. The uncertainty in the spectral radiance of the strip lamps calibrated by NBS at this wavelength is about 2%. The peak of the 247.9 nm CI line is actually calibrated in two steps: first, the continuum emitted by the arc mixture at 250 nm is measured and calibrated at low spectral resolution using the tungsten strip lamp and the air-path predisperser and monochromator; and second, the peak of the carbon line is calibrated with respect to this continuum using the VUV instrument at high resolution. The difference in the VUV system efficiencies between 247.9 and 250 nm is minimal but is measured with the hydrogen arc and taken into account. Care is also taken to ensure that the contribution of the line wing at 250 nm in the low resolution air system is negligible. For a 4.7 nm diameter arc at 60 A the axis temperature of the argon plasma with admixtures of N, C, O, and H was measured to be 11,900 K. A temperature uncertainty of ±160 K is estimated due to an estimated rms uncertainty of about 6% in the blackbody intensity determination at 247.9 nm. This results in an uncertainty of 10% in the blackbody line radiances between 115 and 140 nm.

Besides the temperature of the plasma, the character of the blackbody limited lines is also critical. For example, if the boundary layers between the mixed plasma and the pure argon buffer are too thick, the radiation transfer may be complicated by the density and temperature gradients through the layers. Such conditions are more the rule than exception and are easily detected at high resolution as structure in the line peaks. However, by appropriate flow rate adjustments, this effect can be minimized so that the blackbody plateaus are essentially flat-topped. Referring to figure 6, we see that for a high power hydrogen arc the calibrated spectral radiances obtained using blackbody limited lines are consistent with the corresponding calculated quantities, as was noted in section 2.1.1.

### 2.2 Secondary Standards

#### 2.2.1 Argon Mini-Arc

##### Physical Principles

After development of the hydrogen arc as a primary standard of radiance in the VUV, it was realized that a simpler, portable, more easily operated, and lower powered transfer standard would be of great value. The hydrogen arc is complicated and difficult to operate, and requires a massive highly stable dc power source rated at 1200V and 110A. A secondary standard which could be compared with our primary standard and then transported to the user’s laboratory was recognized as being essential.

The most widely used transfer standard at that time was the commercially available deuterium lamp. Although the deuterium lamp has attractive properties such as its relatively strong continuum, low power requirements, and small size, it also has limitations. First, its region of applicability as a radiometric standard is restricted to wavelengths above 165 nm due to the presence of a many-line molecular deuterium band system below 165 nm. Second, the lamp exhibits a variability related to the positioning of the discharge on the electrodes following each ignition. Finally, the deuterium lamp has aging characteristics which are not well known. At the least, this means that the lamp must be frequently recalibrated to ensure accuracy.

To meet the lack of adequate transfer standards in the far UV, the argon mini-arc was developed at NBS [14]. This source can be conveniently applied as a radiometric transfer standard without the limitations of the deuterium lamp. The wavelength range of the argon mini-arc overlaps the lower range of the tungsten strip lamp in the near UV and extends beyond the short wavelength limit of the low pressure deuterium lamp at 165 nm. The mini-arc was designed to fulfill, insofar as possible, the following goals: 1) an intense line-free continuous spectrum between 115 and 330 nm; 2) stability and reproducibility over many hours of operation; 3) light source and power supply both portable; 4) uniform output over a large solid angle; 5) radiant power output adjustable over a range of several decades; and 6) simple alignment and operation. We now proceed to discussions of the arc construction and operating characteristics.

##### Description of the Arc

**Figure 7:**
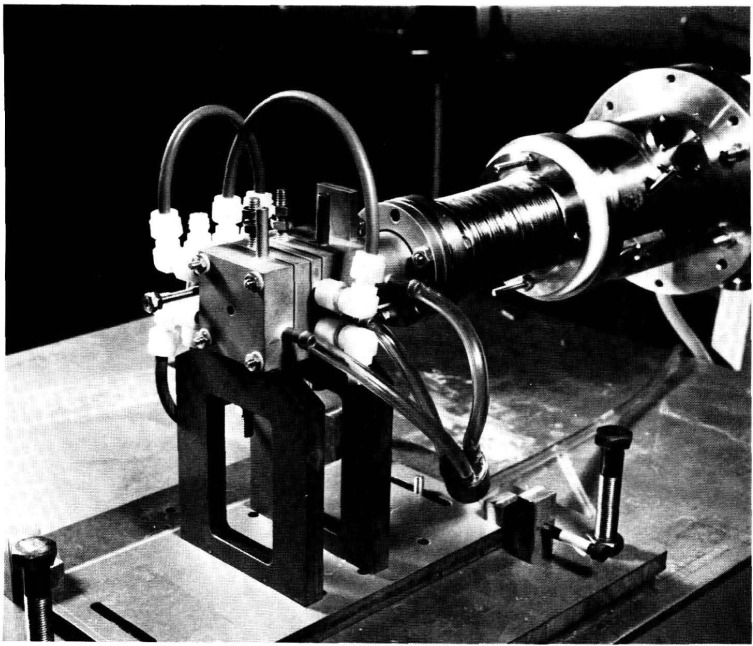
A photograph of the argon mini-arc mated to a monochromator.

Figure 7 is a photograph of the arc source. The model described here was designed specifically for use as a secondary radiance standard and is different in some respects from arc sources previously designed for other purposes. It was developed after experimentation as the simplest model which meets the requirements listed above. Argon was chosen as the operating gas because of its suitability in providing a stable discharge and an intense, line-free UV continuum with minimal power requirements. In the photograph the arc is mated to a monochromator with a stainless steel bellows. Water cooling connections and a triple-tube manifold for supplying argon are also shown. The two vertical threaded posts on the top of the arc are the electrical connections to the anode and cathode. The arc is mounted on an adjustable table which allows precise translation and rotation about two axes. All sources utilized in the NBS VUV radiometry program are mounted on this type of table.

**Figure 8:**
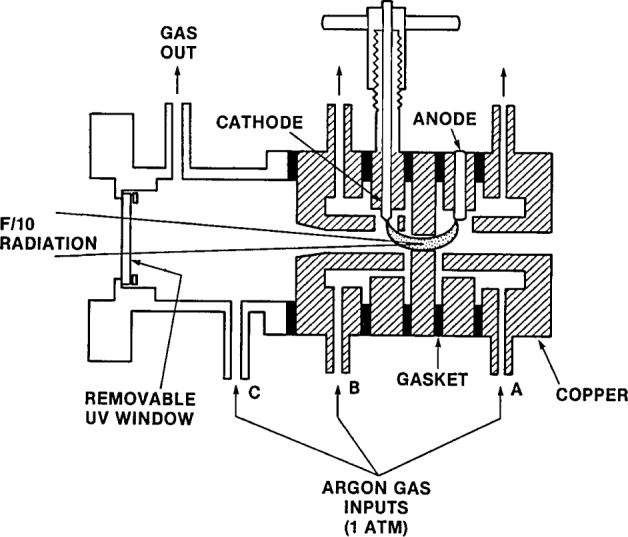
A schematic of the argon mini-arc light source.

As shown in figure 8, which is drawn to scale, the arc source is constructed essentially of five copper plates separated by silicone rubber insulating rings and clamped together to form the device. The central plate forms a channel which guides and constricts the discharge. The plates adjacent to the central plate contain the anode, a 3.2-mm diameter thoriated tungsten rod pressed into its plate, and the cathode, also of tungsten but mounted so that it can be moved into and out of the arc channel. The cathode was made adjustable for ease in igniting the arc. All five plates are water-cooled with the water flowing inside each piece through holes drilled in the sides. These holes are seen in the photograph but not in the diagram. The arc constricting section is 6.3 mm thick with a 4.0 mm diameter hole. Diameters smaller than this were considered unacceptable since they caused a higher plasma temperature and significant Ar II line emission in the wavelength region of interest. Larger diameters were rejected since the radiant power for a given current decreased considerably. The arc plasma is observed end-on through the holes in the electrode pieces, and the radiation calibrated is that emitted along the arc axis and emerging through the magnesium fluoride window on the cathode side. This window is set back to avoid possible contamination from the discharge. The gas purity necessary to maintain arc stability and reproducibility of the continuum emission is ensured by operating with a continuous flow of argon. Maintaining a high degree of argon purity within the arc chamber also minimizes the radiant power of atomic resonance lines in the spectrum which arise from small (ppm) concentrations of oxygen, nitrogen, carbon, and hydrogen. These elements are present due to air and water vapor in the arc chamber and gas handling system. The positions of the gas inlet and outlet ports are chosen to keep all sections of the arc chamber thoroughly purged. The argon is admitted to the inlet ports through plastic tubing connected to a flowmeter. Under the flow rate used here, the pressure in the chamber is unchanged from the ambient atmospheric pressure. The arc may be operated most conveniently with the outlet ports open and the data given below used to account for local and temporal differences in atmospheric pressure, if necessary.

Optical alignment can be performed by sighting with a telescope or laser beam down the arc axis. The distance for proper focus should be measured to the center of the middle copper piece.

The arc discharge is initiated by applying voltage between the electrodes and then inserting a tungsten rod, which is externally connected to the anode potential, until it touches the cathode protruding into the arc channel. The discharge is transferred from the tip of the rod to the anode as the rod is withdrawn. Finally, the cathode is withdrawn from the channel as shown in figure 8. Power for the discharge is furnished by a current regulated supply, the size of which is determined by the current required. For example, the power supply used in some of our experiments was a 1.2 kW, high efficiency switching regulator weighing only 10 kg. For starting the arc without difficulty, it was found necessary to use a ballast resistor of about 0.5 Ω in series and to apply a potential of at least 40 V. The arc can be started at currents above 10 A, and after ignition the resistor may be shorted out if desired. For currents above 10 A the voltage drop across the arc is nearly constant at 28 V.

Wall-stabilized arcs employed for end-on measurements usually are constructed of many constricting plates or disks, in order to obtain a nearly homogeneous plasma extending along the axis to the boundary regions near the electrodes at each end. These boundary regions emit a negligible part of the radiation emerging along the arc axis. The hydrogen and blackbody arcs which we use for primary radiance calibrations are necessarily of this type, since the lengths of the emitting plasma columns must be well defined. In such cases, however, the solid angle over which uniform radiation can be observed is small (f/200 is used for calibrations with the hydrogen and blackbody arcs) and not convenient for the calibration of many optical systems. For a transfer standard, on the other hand, the arc length does not need to be precisely known as long as it is a reproducible quantity. Thus, in order to provide a much greater solid angle, the argon mini-arc was made with only one constricting plate. Measurements show that this construction provides a rather large angular beam of radiation (f/10) having a practically uniform intensity distribution [14]. This should be considered an advantage even if one does not require such a large solid angle, since having the larger available angle makes the angular alignment of the mini-arc less critical.

##### Spectrum

**Figure 9:**
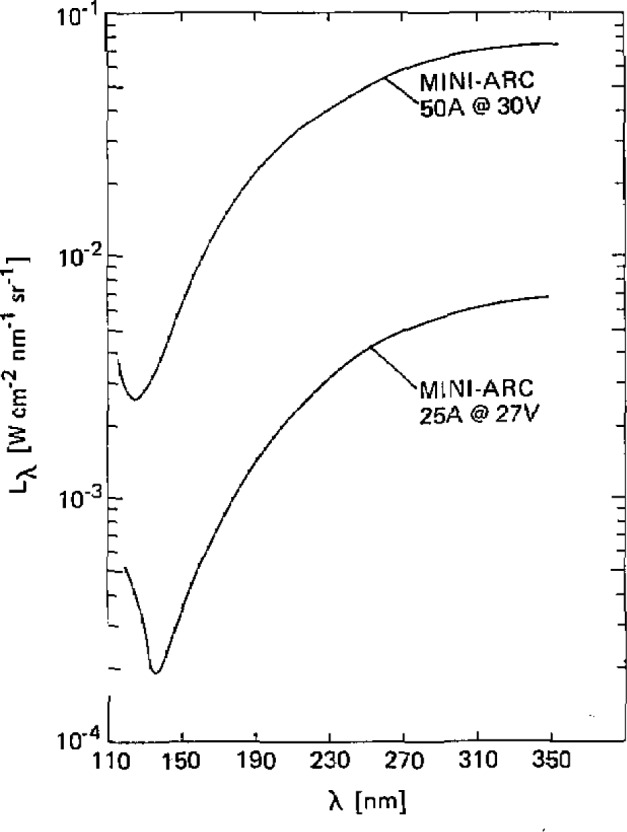
Spectral radiance as a function of wavelength for a mini-arc with an arc plate diameter of 4.0 mm operated at two different currents: 50.0 and 25.0 A.

Figure 9 illustrates the spectral radiance of the argon mini-arc light source for two arc currents: 25.0 and 50.0 A. The continuous spectrum of the argon arc arises primarily from atomic recombination radiation. Near the short wavelength end the molecular continuum mentioned above contributes to some degree, and below 125 nm the increased output is from the wings of the argon resonance lines at 106.7 and 104.8 nm. Below the MgF_2_ window cutoff, one should expect to have strong emission on the wings of the resonance lines with complete absorption at their centers. Below 80 nm there should be complete resonance continuum absorption by ground state argon atoms.

The spectral radiance was determined by direct comparison to the NBS wall-stabilized hydrogen arc [1] between 130 and 330 nm and to a plasma blackbody line radiator below 130 nm [1,3,11]. Data were taken with a spectral resolution of 0.01 nm. The temperature of the blackbody line radiator was determined to be 11,800±100 K by directly measuring the blackbody ceilings at 193, 174, 165, 149, and 146 nm, using the hydrogen arc as a primary standard of spectral radiance. With this temperature the radiant power of other blackbody limited lines emitted below 130 nm could then be calculated. For the calibration of longer wavelengths (>210 nm), the second-order spectrum from shorter wavelengths was eliminated by purging the small volume between the arc and the spectrometer with oxygen. Below 210 nm, argon was used as the purge gas.

The spectral radiance values illustrated in figure 9 and listed in table 2 apply to the plasma radiation emitted along the axis of the mini-arc which is imaged with a focusing mirror on a 0.30 mm diameter aperture (magnification= 1) at a solid angle V200. The basic uncertainty in the spectral radiance is ±5% above 140 nm and ±10% below 140 nm due to uncertainties in the primary radiometric standards. Also included in table 2 for reference purposes is the measured transmission of the MgF_2_ window used on the mini-arc. If one wishes to know the spectral radiance of the plasma itself, the figures in the table must be divided by the transmission. Known systematic deviations or uncertainties in the radiant power due to different operating or imaging conditions are described elsewhere [14].

**Figure 10:**
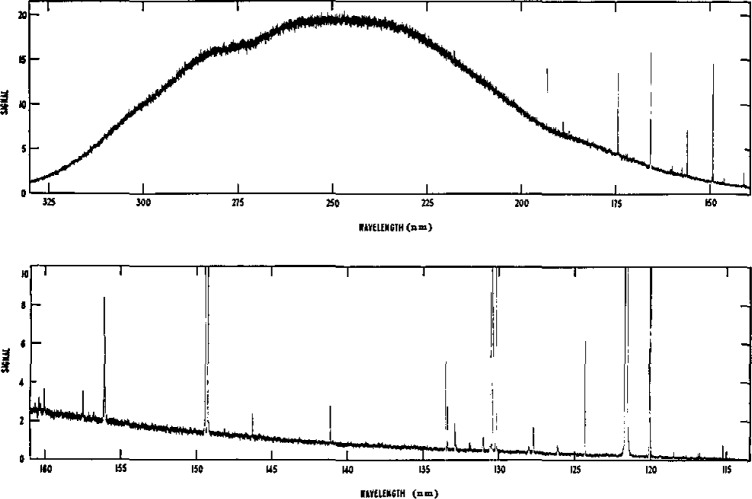
A photoelectric scan of the spectrum of the argon mini-arc between 115 and 320 nm, taken with a 0.01 nm spectral resolution and with a solar blind phototube detector.

There are no Ar I lines in the spectrum between 114 and 330 nm. Below 200 nm there are several narrow Ar II lines and atomic nitrogen, carbon, and oxygen lines in the mini-arc spectrum due to air impurities in the argon gas (99.999% pure). In addition the hydrogen Lyman α line at 121.6 nm is present due to trace quantities of water vapor throughout the system. These impurity lines can be seen in figure 10 which is a photoelectric scan of the spectrum between 115 and 320 nm. The halfwidths of the lines are on the order of 0.01 nm, and the lines are well separated. Their presence has no significant influence on the ability to make absolute continuum measurements except when extremely coarse wavelength resolution is used, as would be the case, for example, if the monochromator were replaced by relatively wideband filters. All lines observed in the spectrum are listed in table 3. From this list one may readily determine if, for a given wavelength and bandpass, one is free of lines. This precaution is necessary since the amount of trace impurities in the arc and the corresponding line intensities may vary somewhat from one system to another.

**Figure 11:**
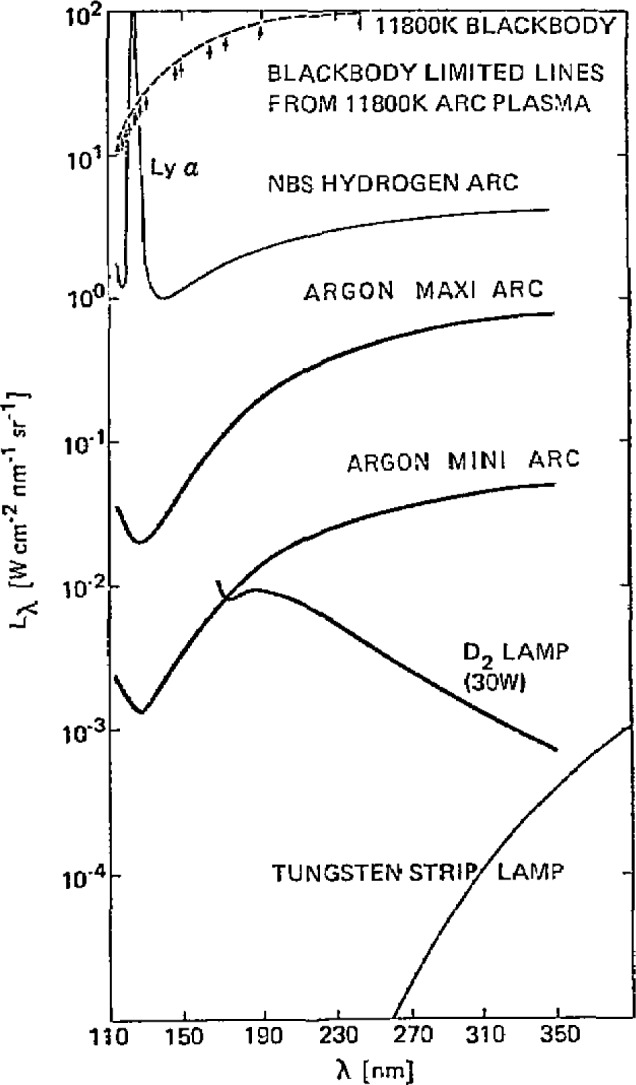
Comparison of the spectral radiance of several UV primary and transfer standard sources.

Figure 11 compares the spectrum of the mini-arc with several other light sources which have been applied as spectral radiance standards. Included in this figure is a representation of the spectra of the two primary standards, the hydrogen wall-stabilized arc and the blackbody line radiator, which were used to calibrate the mini-arc spectrum. The figure clearly illustrates the main advantages of the new transfer source. The mini-arc has substantially larger output and longer UV wavelength range than either the deuterium lamp or the tungsten strip lamp. Also, scattered visible light is not a significant factor, as it is when the tungsten lamp is used as a UV light source. The visible light from the argon arc is of the same order of magnitude as the near UV light, and there is less than a factor of 100 difference between the radiance at 110 and at 330 nm.

#### 2.2.2 Argon Maxi Arc

A recent advance in the arc standards program at NBS is the development of the argon maxi-arc. This source is essentially a high powered version of the mini-arc with three or four arc confining plates instead of one and power ratings of 5–10 kW instead of 1.5 kW. The maxi-arc with three plates has an irradiance approximately 30 times that of a mini-arc and was designed to enable calibrations to be performed at a level comparable to the solar irradiance in the near UV (250–350 nm). The discussion of the mini-arc given above generally applies also to maxi-arcs except for the powers and radiances. Figure 11 gives a comparison of the spectral radiance of the maxi-arc with that of several other UV primary and transfer standard sources. Several of these arcs have been supplied for use in calibration of space experiments.

#### 2.2.3 Deuterium Arc Lamp

##### Introduction

A source which has advantageous properties as a radiometric standard and is quite convenient to use is the molecular deuterium arc lamp [15]. This lamp has considerable UV radiant power, is light and compact, is low-powered (30 W), and with maximum radiant power at 190 nm has a very favorable ratio of UV to visible radiant power. This last fact is important in avoiding systematic errors due to visible scattered light effects when making measurements in the UV. Its disadvantages are that it is restricted to wavelengths above 165 nm due to the presence of a many-line molecular band system below 165 nm; it may exhibit a variability in the positioning of the discharge on the electrodes following each ignition; and its aging characteristics are not well known. On balance, however, the advantages of the deuterium lamp outweigh its disadvantages making it an essential component of any program of UV radiometry. The following discussion will describe the calibration of the deuterium lamp for spectral irradiance in the range 167–350 nm.

Discussion of the spectral radiance calibration of the deuterium lamp is not presented here since it is not a standard calibration service offered by NBS. However, this type of calibration can be requested under the, category “Special Tests of Radiometric Devices in the Near and Vacuum Ultraviolet.”

##### Description and Operation of the Lamp

**Figure 12:**
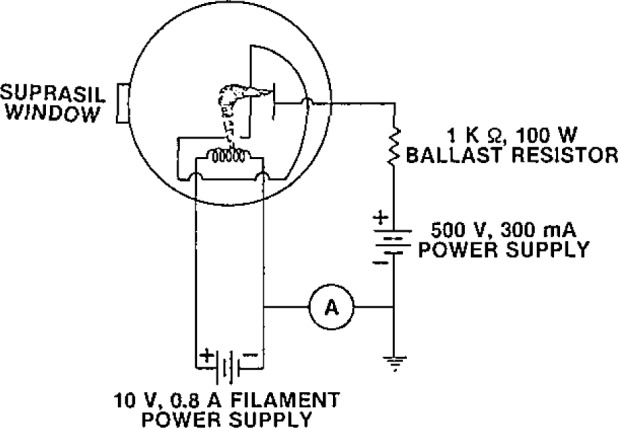
Schematic illustrating the operation of a deuterium lamp. The radiation is measured through the Suprasil window sealed to the quartz lamp envelope.

A schematic illustrating the operation of the deuterium lamp is shown in figure 12. In order to start the lamp, the cathode coil is first heated for 5 s by a dc power supply (10 V at 0.8 A) in order to provide some free electrons which facilitate initiating the discharge. When a voltage of about 400 V is applied to the lamp, an arc forms between cathode and anode in the general form of an L. Most of the UV light is generated at the constricting aperture (1-mm diameter in our case) located in front of the anode. The main dc power supply is a 500 V, 300-mA constant current supply, with 0.1% current regulation. A ballast resistor (1 kΩ, 100 W) is used in the anode circuit because most power supplies cannot react fast enough to maintain a stable arc with only the lamp in the circuit. After the arc is started, the voltage across the arc drops to about 100 V. At this point the heater current is switched off, and the lamp output stabilizes in 20 minutes or less. If the lamp is switched off, it should be allowed to cool back to room temperature before restarting.

## 3. Calibration Methods

### 3.1 Radiance Calibrations

#### 3.1.1 Introduction

Spectral radiance, *L_λ_*, is defined in the Introduction as the radiant power emitted by a source per area per solid angle per wavelength interval: *L_λ_* = [W cm^−2^ sr^−1^ nm^−1^]. A calibration of radiance is performed by directly comparing the source to be calibrated with a standard radiance source. An optical system including lenses and/or mirrors is used to limit the geometrical quantities which are area and solid angle and to direct the radiation to a detector. A monochromator selects the wavelength band desired. First, one source is placed in position and signals are measured at the various wavelengths. This source is then replaced by the second source and the measurements are repeated. The radiance of the source to be calibrated is then obtained from the ratio of the signals times the radiance of the standard source. In general the signal obtained from a source is

$$S(A)=\epsilon_{\lambda }\left(\frac{A}{W}\right)\cdot L_{\lambda }\left(\frac{W}{{\text{cm}}^{2}\text{sr nm}}\right)\cdot A({\text{cm}}^{2})\cdot \Omega (\text{sr})\cdot \Delta \lambda (\text{nm}),$$ (1)

where *A* is the area, Ω the solid angle, Δλ the wavelength bandpass, *L*_λ_ the spectral radiance, and ϵ_λ_ the efficiency of the detection system. If subscript R refers to the standard source and subscript x refers to the one to be calibrated, then

$$\frac{S_{x}}{S_{R}}=\frac{\epsilon L_{\lambda ,x}\cdot A_{x}\cdot \Omega_{x}\cdot \Delta \lambda_{x}}{\epsilon L_{\lambda ,R}\cdot A_{R}\cdot \Omega_{R}\cdot \Delta \lambda_{R}}$$ (2)

If *A*, Ω, and Δλ are identical for each source, then

$$L_{\lambda ,x}=\left(\frac{S_{x}}{S_{R}}\right)\cdot L_{\lambda ,R}.$$ (3)

#### 3.1.2 Argon Arc

The hydrogen arc primary standard is generally used to calibrate an argon arc. After measuring its signals, the hydrogen arc is removed and replaced with the arc to be calibrated. A telescope is used to align the argon arc, and once it is operating, a fine adjustment in position is accomplished by translating the arc both horizontally and vertically to maximize the signal. As with the hydrogen arc, there is a volume between the arc source and the vacuum window. This volume is purged with argon at a flow rate of 5 L/min. The purging must continue for at least 10 min before measurements are begun in order to sufficiently displace the air from this region. This flow rate is continued during the entire calibration. By monitoring the signal at 170 nm where air absorption is strong, the rise in signal during purging may be observed, and the time at which essentially all air is displaced can be determined. There are two cases to consider: 1) if the arc to be calibrated contains a window, the purge region is isolated from the arc, and any purge rate which is sufficient to maintain the region free of air may be used; and 2) if the arc to be calibrated is one without a window, the flow in the “purge” region is not independent of the arc, and the same flow arrangement must be used whenever the arc is used as a secondary standard. This is necessary because the flow rate in the purge region can have an effect on the arc operation and hence on the radiance of the arc. Once the argon arc is operating and the purge region is clear of all air, signals are measured for 124 < λ < 360 nm, the range of the hydrogen arc. In addition to the wavelengths covered by the hydrogen arc, signals are also measured at shorter wavelengths extending to 115 nm. In this region, however, there are numerous lines in the spectrum from minute air impurities. These lines are typically resonance lines and are extremely strong even at very low concentrations. The wavelengths at which the continuum is measured are selected to avoid interference from these relatively strong lines. Also, in order to avoid interference from the lines, the wavelength bandpass must be no greater than 0.2 nm (700 μm slits on the 3 m monochromator). The continuum from this short wavelength region is calibrated using a second primary standard, the blackbody line arc.

#### 3.1.3 Blackbody Line Arc

Another primary standard source, the blackbody line arc, is used to extend the calibration to wavelengths below 124 nm. This is also a wall-stabilized arc, operated with argon but including small admixtures of H, O, C, N, and Kr. These admixture elements furnish spectral lines which can be made to be *optically thick*, i.e., they reach a maximum radiance value at the wavelength of the line.

#### 3.1.4 Tungsten Lamp Standard

In the calibration of radiance one additional standard source, the tungsten strip lamp [10], is applied. The tungsten lamp is used for two reasons: 1) to increase the accuracy of the calibration, and 2) to extend the calibration to longer wavelengths. The wavelength ranges of the hydrogen arc and tungsten lamp overlap in the near UV region, and although the uncertainty in the radiance of the hydrogen arc is ±5%, tungsten lamps are calibrated for radiance with an uncertainty of ±2.3% in the near UV. Therefore, the calibration of a source extending to the near UV may be increased in accuracy by comparing it directly to the tungsten strip lamp. If this is done, a calibration using the hydrogen and blackbody line arcs is considered to furnish only the relative spectral distribution of the radiance of the unknown source. A calibration with the tungsten lamp at the long wavelength end of the range of the hydrogen arc then serves to set the absolute scale of the radiance.

#### 3.1.5 Calibration of Argon Arcs Relative to an NBS Argon Arc

For most calibrations the hydrogen arc and blackbody line arc are not employed, but rather calibrations are performed using a mini-arc (the NBS argon arc) as a transfer standard. The transfer standard is employed since calibrations with the hydrogen and blackbody arcs are much more difficult and time consuming than calibrations performed relative to an argon arc. Moreover, the comparison of two similar sources (two argon arcs) can be accomplished in reasonable time with less error than is involved in a calibration involving the hydrogen and blackbody line arcs. Therefore only a slight decrease in accuracy results from employing the transfer standard. The calibration of one argon arc relative to another proceeds similarly to the calibration of an argon arc relative to the hydrogen arc. The NBS argon arc is operated without a window using a purge rate of 5 L/min. The arc to be calibrated usually contains a window, and thus the calibration of the arc must include a separate measurement of its transmission.

#### 3.1.6 Argon Mini-Arc Spectral Radiance Standard

An argon mini-arc has been designed, tested, and operated as a transfer source of spectral radiance for the wavelength range from 114 to 330 nm. Calibration has been performed using two primary standard sources: the hydrogen arc from 130 to 330 nm and the blackbody line radiator from 114 to 130 nm. The mini-arc has the following principal features: 1) a steady-state reproducible source with dc power requirements of less than 1.5 kW; 2) an intense continuum which is line-free between 194 and 330 nm and which has only a few narrow and widely spaced impurity lines between 114 and 194 nm; 3) a uniform output over a solid angle as large as f/9; 4) negligible aging effects over at least a 24 hour period; and 5) uncertainties (2*σ*) in the absolute spectral radiance of 5.3% above 140 nm and 10. 1% between 114 and 140 nm. The radiant power emitted by the mini-arc is influenced primarily by the arc diameter, the arc current, and the transmission of the UV window material.

### 3.2 Irradiance Calibrations

#### 3.2.1 Establishment of Irradiance Scale: General Method

Spectral irradiance is the radiant power incident upon a target area per area per wavelength band: *E* = [W cm^−2^ nm^−1^]. A source of radiation may serve as a standard source of irradiance by operating it at a given distance from the target area. If one had a radiance source with uniform radiance over its emitting area, the irradiance at a given distance from this source could be easily computed. In general however, radiance sources are non-homogeneous, and the radiance is known only for a small portion of the source, i.e., an area element which can be approximated to be homogeneous. Thus, the irradiance at some distance from the source can be determined only if radiation from outside the calibrated area is prevented from reaching the measurement system. This can be done by the use of optical imaging and/or collimating apertures. For applications in the VUV, the method using collimating apertures [16] has proved to be more practical and is the basis for the measurements described here.

The collimating aperture method is as follows. A radiation source that is homogeneous over a certain emitting area and whose spectral radiance has been previously determined is situated a given distance from a monochromator. A pair of apertures, one at the entrance slit of the monochromator (the field aperture) and the other as close to the source as possible (the source aperture), is chosen so that only the radiation from the homogeneous portion of the source is measured. The spectral irradiance at the field aperture is given by the product of the known spectral radiance of the source and a geometric factor dependent on the aperture dimensions and their locations relative to the source. This geometric factor contains the information on the effective area of the emitting source, the solid angle of the radiation beam incident upon the field aperture, and the irradiated area.

In principle, these quantities can be determined by measurement. However, a direct determination of the geometric factor is unnecessary, as is shown by the following discussion. The response of a spectroradiometer as a function of wavelength to a suitably collimated radiance standard is essentially a measure of the system detection efficiency on a relative scale. If the same spectroradionmeter is irradiated with an unknown source and the response is measured again, the spectral irradiance of the unknown source can be determined, also on a relative scale. Then, provided that the wavelength range of calibration extends to the visible or near-UV region in which standard sources of irradiance are available, the absolute spectral irradiance of the unknown source can be determined at one wavelength within the calibrated wavelength range. This absolute value can then be used to normalize the relative scale of irradiance to an absolute scale.

#### 3.2.2 Argon Arc Calibrations

Two types of sources are calibrated as irradiance standards, the argon mini-arc and the deuterium lamp. Calibration of an argon arc will be discussed first.

The light source used here as a standard of spectral radiance in the VUV and near UV is an argon mini-arc previously calibrated by the hydrogen arc. This source is used according to the method described above to calibrate a second mini-arc as an irradiance standard. The measurements in the VUV spectral range are performed using a SEYA monochromator. The above measurements determine the irradiance of the unknown source only on a relative scale. To obtain an absolute scale, the irradiance of the unknown source is compared in the near UV region to that of a calibrated tungsten-quartz-halogen irradiance standard [17]. This measurement is carried out on a double monochromator, with an integrating sphere used as its entrance aperture. For this measurement this setup has two advantages. First, the double monochromator greatly reduces scattered light which otherwise would be a serious problem for the quartz-halogen source. Second, the integrating sphere serves as a better method of rendering the signal insensitive to the direction of radiation from the source. This is important in comparing the small sized arc source with the much larger quartz-halogen source area.

The determinations of spectral irradiance are based on the assumptions that the system efficiencies of the measuring spectroradiometer are independent of the angle at which the radiation enters and that diffraction effects are not significant. Since the monochromator grating used must be assumed to have a nonuniform reflection efficiency, a diffuser located directly behind the field aperture is used to ensure that the first assumption is met. A magnesium fluoride window, ground on one side, was used as the diffusing element. Although it cannot be expected to be as good a diffuser as an integrating sphere, it was shown to be suitable, at least over a relatively small range of angles. For the argon mini-arc the uncertainty in the spectral irradiance that is due to the nonideal properties of the diffusing window can be as high as 6%.

The possible effects of diffraction must also be considered. For an extended homogeneous radiation source whose dimensions are large compared with the source aperture, it can be shown that Frauenhofer diffraction effects introduce no wavelength dependence and that the geometric factors are wavelength independent. This can be understood qualitatively by realizing that some percentage of the radiation from each radiating point does not pass through the field aperture because of diffraction. However, a complementary point can always be found in 'the domain of the extended homogeneous source that exactly makes up for such a loss. By substituting a different-sized aperture we have shown that the inhomogeneity of the mini-arc is insufficient to cause any measurable effects due to diffraction.

The vacuum spectroradiometer consists of a solar blind photonmultiplier and a spectrometer with a 2-mm diameter field aperture. This aperture is mounted on a MgF_2_ diffusing window located 50 mm in front of the entrance slit. The diffuser was located some distance in front of the entrance slit so as not to overfill the grating and thereby increase the scattered light, which was less than 2% of the weakest signal. The mini-arc was located 50 cm from the field stop, and a 0.3 mm-diameter aperture placed 5 cm from the mini-arc center was used to restrict the size of the radiating area to about 0.5 mm in diameter. The 2*σ* uncertainty in the absolute spectral radiance of the mini-arc has been given above to be 5.3% between 140 and 330 nm.

**Figure 13:**
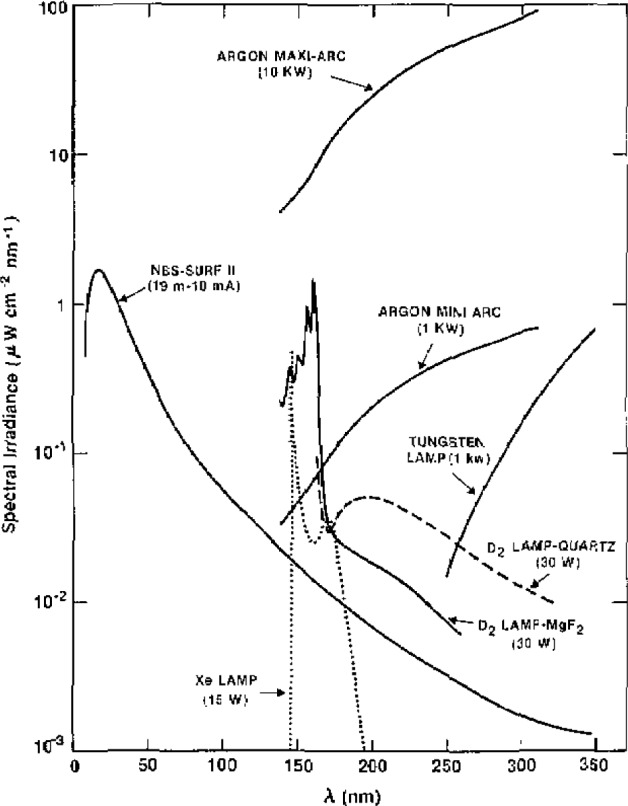
Absolute spectral irradiance measured as a function of wavelength at a distance of 50 cm from the field aperture for five different continuum sources with the indicated power requirements. The spectrum of the D_2_ lamp with MgF_2_ window below 170 nm, measured for a 1 nm bandpass, is a pseudo-continuum made up of blended lines. Shown for comparison purposes are spectra of the 250 MeV National Bureau of Standards synchrotron radiation facility, for a beam current of 10 mA and a field aperture distance of 19 m, and the tungsten-quartz-halogen lamp, for the standard 50-cm distance.

The total uncertainty in the irradiance calibration of an argon mini-arc, including uncertainties in the primary source calibration and the transfer procedure, is estimated to be ⪅ 10%. Although the arc emits radiation at shorter wavelengths than that shown in figure 13, the short wavelength limit is taken to be 140 nm because of the low signal obtained from the stopped-down mini-arc radiance standard at shorter wavelengths. The bandpass for the radiance-to-irradiance transfer here was set at 1 nm. Not shown in figure 13 are several lines in the argon arc spectrum that are due to residual gas impurities [14]. The irradiance of the mini-arc was put on an absolute scale by determining its absolute spectral irradiance at 280 nm, using a tungsten-quartz-halogen lamp as an irradiance standard. A separate spectroradiometer utilizing a BaSO_4_-coated integrating sphere, a predispersing monochromator, and an analyzing spectrometer was used for this measurement. One measurement of this type is sufficient to determine the absolute irradiance at all wavelengths [16].

#### 3.2.3 Deuterium Lamp Calibration

Deuterium lamps are calibrated as spectral irradiance standards in the near UV range 200⩽λ⩽350 nm by another calibration group in the Radiometric Physics Division of NBS. The lamps calibrated are of the side-on type. Prior to calibration they are potted in a base identical to that of the tungsten-quartz-halogen lamps. Our calibrations of such lamps usually consist of extending the spectral range in the vacuum UV down to 165 nm. The measurements are performed on the SEYA monochromator setup, using the same method as was described in the previous section for calibrating an argon arc as an irradiance source.

Briefly, a set of commercially available deuterium lamps was calibrated for spectral irradiance in the 167–350 nm spectral range. At 250 nm and above the spectral irradiance values were obtained using a tungsten-quartz-halogen lamp whose calibration is based on an NBS blackbody. Below 250 nm the relative spectral distribution of the deuterium lamps was determined through the use of an argon mini-arc spectral radiance transfer standard whose calibration is based upon the NBS wall-stabilized hydrogen arc. The absolute values assigned to the deuterium lamps were then obtained by normalizing to the spectral irradiance values at 250 nm and above. Confidence in this procedure was established by comparing the absolute spectral irradiance of the deuterium lamps as determined independently by the argon mini-arc radiance standard and the tungsten-quartz-halogen irradiance standard in the 250–330 nm range. The agreement was within 3%. The large UV flux and high stability of the mini-arc are the properties which make possible the calibration below 250 nm.

Since the deuterium lamps are aligned and potted in bipost bases identical to those used in mounting tungsten-quartz-halogen lamps, the two standards are interchangeable in a given optical system. Because the shapes of the spectral distributions of these lamps are so different, the presence of systematic errors in a measurement system may be detected by intercomparing the two sources. The spectral irradiance of the 30 W deuterium lamp is equal to that of the 1000 W quartz-halogen lamp at about 260 nm, 100 times stronger at 200 nm, and 100 times weaker at 350 nm.

The uncertainties in the absolute values of the deuterium lamp spectral irradiance are: 200 nm and above, 6%; 171 to 195 nm, 7%; and 170 nm and below, 10%. A major contribution to each uncertainty is the variability associated with the striking of the deuterium lamps. As a result all calibrated deuterium lamps supplied to customers by NBS are preselected for variabilities of 4% or less. For higher accuracy and confidence, one may take advantage of the result that the relative spectral distribution of the deuterium lamps is reproducible to within about 1%. Thus, the uncertainty may be reduced by renormalizing the absolute scale with a quartz-halogen lamp after each ignition to eliminate any variability in the strike. This procedure makes possible a reduction in the uncertainties of about 3%.

Additional work has shown that under certain conditions the deuterium lamp with an MgF_2_ window may be used as a radiometric standard down to 115 nm [18]. This effort was directed at determining the circumstances under which portions of the many-line molecular spectrum below 167 nm can be used as a continuum.

## Figures and Tables

**Table 1:** Maximum emission coefficient from a 1-atm hydrogen plasma in LTE calculated as a function of wavelength^a^

λ(nm)	*ϵ*(W cm^−3^ nm^−1^ sr^−1^)	*T*(K)	$\frac{\text{Ly}\alpha }{\epsilon }(\%)$
350	0.73	17,000	0
300	0.68	17,000	0
250	0.59	17,500	0.01
200	0.424	18,500	0.07
190	0.386	18,800	0.11
180	0.347	19,200	0.20
170	0.308	19,500	0.34
160	0.269	20,000	0.65
150	0.232	21,000	1.3
140	0.200	21,700	4
130	0.245	17,500	54
128	0.372	16,500	77
126	0.79	16,500	90
124	3.22	16,000	98

a The percentage of this emission due to Ly α and she arc temperature required for maximum emission are also listed.

**Table 2:** Spectral radiance as a function of wavelength for a 50.0-A argon mini-arc light source with an arc plate diameter of 4.0 mm^a^

λ(nm)	*L*_λ_(mW cm^−2^ nm^−1^ sr^−1^)	*T*	λ(nm)	*L*_λ_(mW cm^−2^ nm^−1^ sr^−1^)	*T*
330.5	70	0.945	198.0	26.6	0.900
325.5	70	0.943	192.5	24.0	0.900
320.5	69	0.942	191.0	23.5	0.894
315.5	69	0.942	186.0	20.8	0.891
310.5	68	0.941	181.0	18.4	0.883
305.5	67	0.940	176.0	16.1	0.873
300.5	66	0.939	173.5	15.0	0.870
295.5	64	0.939	170.0	13.6	0.860
290.5	63	0.938	166.5	11.8	0.850
285.5	62	0.938	165.0	11.1	0.848
280.5	60	0.938	162.0	9.9	0.840
275.5	58	0.938	159.5	9.3	0.826
270.5	57	0.937	157.0	8.3	0.810
265.5	55	0.937	155.0	7.8	0.798
260.5	54	0.937	153.5	7.3	0.785
255.5	52	0.935	151.0	6.5	0.765
250.5	50	0.933	148.5	5.9	0.740
245.5	48.1	0.932	147.0	5.4	0.724
240.5	45.2	0.931	144.0	4.73	0.700
235.5	43.3	0.928	139.5	3.81	0.672
230.5	40.9	0.925	135.0	3.38	0.655
225.5	39.1	0.922	129.5	2.77	0.662
220.5	36.3	0.920	127.0	2.65	0.642
215.5	34.6	0.917	123.5	2.54	0.626
210.5	32.4	0.914	118.5	2.77	0.597
205.5	30.3	0.910	116.0	3.34	0.530
200.5	27.9	0.900	114.5	3.74	0.439

a The measured transmission *T* of the are window is also listed. The radiance of the plasma itself is obtained by calculating the quantity *L*_λ_*T*^−1^.

**Table 3:** Survey of emission lines appearing in spectrum of mini-arc

λ(mn)	Identification	Radiance relative to continuum^a^
115.22	OI	1
116.79	NI	1
117.69	NI	0.3
118.94	CI	0.3
119.38	CI	1
119.99	NI	30
121.57	HI	100
124.33	NI	5
126.13	CI	2
127.75	CI	5
128.98	CI	1
130.22	OI	15
130.55	OI	10
131.07	NI	1
131.95	NI	0.5
132.93	CI	4
133.53	CII	8
135.58	CI	0.2
141.19	NI	0.7
143.19	CI	0.1
145.91	CI	0.1
146.33	CI	1
146.75	CI	0.1
148.18	CI	0.2
149.26	NI	4
149.47	NI	6
156.10	CI	4
157.50	ArII	0.2
160.04	ArII	0.2
160.35	ArII	0.1
160.65	ArII	0.05
165.72	CI	2
174.27	NI	1
174.53	NI	0.5
175.19	CI	0.1
187.31	ArII	0.05
188.90	ArII	0.1
193.09	CI	2
247.86	CI	0.05

a In column three are listed line radiances relative to the continuum, measured with 0.25-nm spectral resolution

## Appendix. NBS Calibration Services in the Near and Vacuum Ultraviolet

### 1. Spectral Irradiance Standard, Argon Mini-Arc (140–330 nm), SP 250 No. 40010S (7.6B)

An argon mini-arc supplied by the customer is calibrated for spectral irradiance at 10 nm intervals in the wavelength region 140–330 nm. Absolute values are obtained by comparing the radiative output with laboratory standards of both spectral irradiance and spectral radiance. The spectral irradiance measurement is made at a distance of 50 cm from the field stop. Uncertainties (2*σ*) are estimated to be less than ±10% in the wavelength region 140–200 nm and within ±6% in the wavelength region 200–330 nm. A measurement of the spectral transmission of the arc window is included in order that the calibration be independent of possible window deterioration or damage.

### 2. Spectral Radiance Standard, Argon Mini-Arc (115–330 nm), SP 250 No. 40020S (7.6C)

The spectral radiance of argon mini-arc radiation sources is determined to within a 2*σ* uncertainty of less than 6% over the wavelength range 140–330 nm and 11% over the wavelength range 115–140 nm. The calibrated area of the 4 mm diameter radiation source is the central 0.3 mm diameter region. Typical values of the spectral radiance are: at 250 nm, *L*_λ_ = 30 mW cm^−2^ nm^−1^ sr^−1^; and at 150 nm, *L*_λ_ = 3 mW cm^−2^ nm^−1^ sr^−1^. The transmission of the demountable MgF_2_ lamp window and that of an additional MgF_2_ window are determined individually so that the user may check periodically for possible long term variations.

### 3. Spectral Irradiance Standard, Deuterium Arc Lamp (165–200 nm), SP 250 No. 40030S (7.6D)

The lamp is calibrated at 10 wavelengths from 165 to 200 nm at a distance of 50 cm. Its spectral irradiances are about 0.05 μW cm^−2^ nm^−1^ at 165 nm, 0.03 μW cm^−2^ nm^−1^ at 170 nm, and 0.05 μW cm^−2^ nm^−1^ at 200 nm. The approximate 2*σ* uncertainty relative to SI units is estimated to be less than 10%. The lamp is normally supplied by NBS and requires 300 mA at about 100 V.

### 4. Special Tests of Radiometric Devices in the Near and Vacuum Ultraviolet, SP 250 No. 40040S (7.6A)

Tests of customer supplied radiometric devices not included above are performed at the direction of the customer.
